# Platelet Contributions to Myocardial Ischemia/Reperfusion Injury

**DOI:** 10.3389/fimmu.2019.01260

**Published:** 2019-06-06

**Authors:** Nancy Schanze, Christoph Bode, Daniel Duerschmied

**Affiliations:** Department of Cardiology and Angiology I, Heart Center, University of Freiburg and Faculty of Medicine, University of Freiburg, Freiburg, Germany

**Keywords:** myocardial infarction, ischemia reperfusion injury, platelets, reperfusion, ischemia

## Abstract

Obstruction of a coronary artery causes ischemia of heart tissue leading to myocardial infarction. Prolonged oxygen deficiency provokes tissue necrosis, which can result in heart failure and death of the patient. Therefore, restoration of coronary blood flow (reperfusion of the ischemic area) by re-canalizing the affected vessel is essential for a better patient outcome. Paradoxically, sudden reperfusion also causes tissue injury, thereby increasing the initial ischemic damage despite restoration of blood flow (=ischemia/reperfusion injury, IRI). Myocardial IRI is a complex event that involves various harmful mechanisms (e.g., production of reactive oxygen species and local increase in calcium ions) as well as inflammatory cells and signals like chemokines and cytokines. An involvement of platelets in the inflammatory reaction associated with IRI was discovered several years ago, but the underlying mechanisms are not yet fully understood. This mini review focusses on platelet contributions to the intricate picture of myocardial IRI. We summarize how upregulation of platelet surface receptors and release of immunomodulatory mediators lead to aggravation of myocardial IRI and subsequent cardiac damage by different mechanisms such as recruitment and activation of immune cells or modification of the cardiac vascular endothelium. In addition, evidence for cardioprotective roles of distinct platelet factors during IRI will be discussed.

## Introduction

Myocardial infarction (MI) is the leading cause of death in the western world (1). The role of platelets in the progression of coronary plaques and the thrombotic occlusion of coronary vessels leading to ischemia and MI is well understood (2). Besides this, microembolization, and platelet accumulation within the affected microcirculation of the myocardium during late ischemia and reperfusion (IR) lead to a secondary tissue damage (2). Rapid restoration of blood flow after MI is the primary goal of state-of-the-art therapy in order to limit the damage of cardiac tissue caused by the lack of oxygen supply. Paradoxically, reperfusion itself also causes adverse effects and increases infarct size (1, 3). This phenomenon is called ischemia/reperfusion injury (IRI) and might account for nearly 50% of the final myocardial damage in acute MI (1). IRI can cause myocardial stunning, the no-reflow phenomenon, reperfusion arrhythmia and also lethal reperfusion injury (reviewed in (4). As underlying mechanisms oxidative stress, calcium overload and pH shifts have been described (1). In this context, mitochondrial damage including disruption of ATP production and opening of the mitochondrial permeability transition pore with subsequent necrotic and apoptotic cell death play an important role (5). Additionally, ischemia–reperfusion induced alterations in the regulation of cytosolic osmolality and cell volume cause cellular and interstitial edema that are associated to microvascular obstruction, cell dysfunction and death (6, 7). Reperfusion has repeatedly been shown to be an inflammatory state that is accompanied by leukocyte infiltration (8, 9). While a certain degree of inflammation is necessary for cardiac repair after IRI it may also develop in an unwanted direction and extend injury (9, 10). Already since the 1980s an involvement of platelets in the inflammatory reaction associated to IRI has been recognized. The damage caused by IR in the vascular endothelium causes activation of circulating platelets, resulting in the release of their immunomodulatory contents (11). The degree of platelet activation is related to the duration of the preceding ischemia and the extent of reperfusion injury (5). This mini review aims to summarize mechanisms of platelet contributions to myocardial IRI.

## The Immunomodulatory Equipment of Platelets

Platelets are small anucleate cells that circulate through the bloodstream in large numbers. Beyond their primary role in hemostasis platelets have been recognized as immunomodulatory cells that fulfill their tasks through a variety of mechanisms ranging from secretable factors to stably or variably expressed surface receptors (12). Specifically, platelets secrete or expose adhesion proteins like fibrinogen, fibronectin, von Willebrand factor, thrombospondin or P-selectin that are involved in cell-cell interactions (e.g., with leukocytes or endothelial cells) during inflammation (13–20). Mitogenic factors released by platelets such as platelet-derived growth factor (PDGF) and transforming growth factor beta (TGFβ) target monocytes, macrophages as well as T-cells, for example to achieve wound healing or immunosuppression (21–24). And chemokines like RANTES/C-C motif ligand (CCL)5, platelet factor 4 (PF4)/ C-X-C motif ligand (CXL)4 or serotonin (5-hydroxytryptamine) are involved in leukocyte recruitment to sites of inflammation (25–29). Moreover, platelets can trigger the complement activation and play a role in localizing inflammatory areas (30). Already with these few examples of immunomodulatory platelet functions in mind, it is obvious to implicate these cells as key contributors to the myocardial IR associated inflammatory response.

## Experimental Models of Ischemia/Reperfusion Injury

In the past years a number of experimental models of IRI were applied in various species by different research groups. The following table briefly summarizes the models mentioned in this review with their respective modes of platelet activation (Table 1).

**Table 1:** Experimental models of ischemia/reperfusion injury.

**Model**	**Species**	**Characteristics**	**Modes of platelet activation**	**References**
LAD ligation	Mouse, rat, rabbit, minipig, and sheep	Intact heart with physiological blood flow Localized ischemia induction via ligation of LAD No occlusive thrombus formation Reperfusion with whole blood via reopening of LAD ligature	Accumulation of procoagulants due to stasis Activated endothelium due to stasis, hypoxia and subsequent reperfusion Cytokines and chemokines released in the ischemic myocardium To some extent by the invasive surgical procedure	(31–44)
Isolated working heart preparations	Guinea pig and rat	Perfusion with physiological cell-free buffer Induction of global ischemia and reperfusion with cell-free buffer Optional perfusion with washed platelets or supernatants of aggregated platelets	Activated endothelium Cytokines, chemokines released in ischemic myocardium Optional pre-activation by chemical agents Optional pre-activation by IRI in AMI patients	(45–48)
Isolated working heart preparations, low flow ischemia	Guinea pig	Perfusion with physiological cell-free buffer Induction of global low flow ischemia (1 ml/min) and subsequent reperfusion Optional perfusion with platelets	Exogenous thrombin activation during low flow ischemia and beginning of reperfusion	(49)
Coronary thrombosis induction	Dog	Coronary thrombus induction by electrolytic injury Localized ischemia Reperfusion by thrombolysis with tissue plasminogen activator	Injured endothelium (e.g., GPVI interaction with exposed subendothelial collagen, PSGL-1/P-selectin interaction etc.) Shear stress Activated endothelium due to ischemia and reperfusion Cytokines, chemokines released in ischemic myocardium	(50)

## Platelet Membrane Proteins in IRI

### Collagen Receptor

The combination of glycoprotein (GP) VI with Fc receptor (FcR)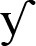 forms the platelet collagen receptor. Takaya et al. demonstrated in 2005 that collagen-dependent activation of platelets can drive the extension of myocardial IRI. In their study, knock-out (k.o.) of FcR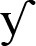 improved outcome of mice after myocardial IR with the infarct size being significantly smaller than in wildtype (WT) mice. Mechanistically the FcR
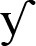
^−/−^ mice displayed less platelet aggregation and occlusive microthrombi, less platelet spleen tyrosine kinase (Syk) activation as well as reduced myeloperoxidase (Mpo) activity in the injured area (51). A more recent study confirmed a role of the collagen receptor in IRI by targeting GPVI with a monoclonal antibody derivative in a murine *in vivo* model of left anterior descending artery (LAD) ligation followed by reperfusion. Anti-GPVI treatment significantly reduced infarct size vs. control, which was again primarily based on an improved microperfusion (52).

### Adenosine Diphosphate (ADP) Receptor

The platelet P2Y_12_ receptor is responsible for mediating the sustained ADP-dependent platelet aggregation response. In a dog coronary thrombosis model, ticagrelor, a reversibly binding P2Y_12_ receptor antagonist, was shown to significantly decrease infarct size and rapidly restore myocardial tissue perfusion by inhibition of ADP-mediated platelet aggregation and recruitment (50). Supporting results were demonstrated by infusing platelets from acute MI (AMI) patients (untreated or treated with 10 μM of the P2Y_12_ inhibitor cangrelor) into isolated rat hearts that were subjected to 40 min of ischemia with subsequent 60 min of reperfusion. Platelets from AMI patients significantly augmented myocardial injury while P2Y_12_ blockage with cangrelor reduced infarct size and attenuated the adverse effects of platelet infusion on cardiac function (48). Besides direct inhibition of aggregation, protective effects of cangrelor during IRI could in part be mediated by inhibition of platelet P-selectin expression and platelet-leukocyte interactions (53). Furthermore, in a rabbit model of myocardial IR, coapplication of individual inhibitors revealed a role for adenosine A_2B_ receptors, ERK, Akt, redox signaling, and mitochondrial KATP channels in mediating the protective cangrelor effect independent of its antiaggregatory properties (37).

### Glycoprotein-IIb/IIIa-Receptor

Given its important role in platelet activation GPIIb/IIIa is predestined as a contributor to IRI. Indeed, inhibition of GPIIb/IIIa reduces platelet induced aggravation of IRI in isolated rat hearts. Infusion of platelets from AMI patients worsened myocardial injury in hearts subjected to IR, as measured by left ventricular (LV) developed pressure, higher maximal LV end-diastolic pressure and coronary resistance as well as increased lactate dehydrogenase (LDH) release and infarct size. Pretreatment of platelets with the GPIIb/IIIa inhibitor abciximab greatly attenuated these effects (48). Mechanistically, in addition to interrupting platelet adherence to the reperfused endothelium through GPIIb/IIIa-fibrinogen binding, abciximab also interferes with other mechanisms of platelet adhesion including the vitronectin receptor or leukocyte Macrophage-1 antigen (Mac-1) (48, 54, 55). In contrast, a publication from 2016 did not confirm a significant effect of GPIIb/IIIa antagonization on infarct size using a specific monoclonal antibody derivative in a murine *in vivo* model of left coronary artery ligation (52). Yet, another study confirmed the adverse effect of GPIIb/IIIa dependent intracoronary platelet retention during low flow ischemia on cardiac function using the GPIIb/IIIa inhibitor tirofiban. This was partially attributed to tirofiban-induced blockage of platelet adherence to the vessel wall mediated by an interaction of the GPIIb/IIIa-receptor and von Willebrand-factor (56). However, in this study no GPIIb/IIIa effect on cardiac function was observed during the reperfusion phase (56), suggesting that conflicting results between studies might at least partially be based on different time points of inhibitor application during the course of IRI, especially since platelet adhesion to reperfused endothelium and platelet-mediated myocardial damage have been described to occur very early after reperfusion (48). Additionally, the use of different experimental models which induce different modes of platelet activation are a decisive factor for experimental outcome (see Table 1).

### P-Selectin

P-selectin is an adhesion molecule expressed on the surface of activated platelets and mediates cell-cell interactions involving platelets, e.g., platelet-neutrophil-complexes which have been associated with many inflammatory conditions (14, 57). Platelet P-selectin expression is increased after IR in several animal models as well as in humans (48, 58). Infusion of platelets activated in this manner into isolated rat hearts subjected to ischemia and reperfusion strongly increases myocardial LDH release representing cardiomyocyte damage (58). In loss of function approaches it was demonstrated that the chemical or small molecule dependent blockade of platelet P-selectin has beneficial effects on platelet mediated reperfusion injury after myocardial IR in pigs and rats, respectively (38, 43). Additionally, a study using genetically modified mice confirmed these results as significantly smaller infarct sizes after myocardial IR were observed in P-selectin k.o. mice or mice transfused with P-selectin k.o. platelets as compared to wild-type (32). Based on these studies the role of activated platelets in the process of myocardial IRI seems to depend at least in part on their activation status as represented by platelet-derived P-selectin. Mechanistically, enhanced P-selectin expression on platelets increases adherence to the reperfused endothelium as well as postischemic leukocyte adhesion, thereby aggravating the inflammatory reaction associated to IRI. However, in terms of leukocyte recruitment it needs to be taken into account that endothelial P-selectin expression can contribute to the observed effects of P-selectin antagonization in some of the reported experimental models, too (59).

### G-Proteins

G proteins are involved in transmitting signals from a variety of stimuli outside of a platelet to its interior. G_α*q*_ k.o. in mice eliminates platelet function in terms of aggregation and secretion of cytokines. In these mice infarct size to area at risk ratio was significantly smaller as compared to WT after 30 min of regional myocardial ischemia (LAD ligation) followed by 24 h of reperfusion. Additionally, G_α*q*_ k.o. improved fractional shortening in this model. The beneficial effects were resembled by transplantation of G_α*q*_ k.o_._ bone marrow into WT mice (34). The effect of G_α*q*_ k.o_._ outperformed the protection of sole inhibition of platelet aggregation and was accompanied by reduced expression of the fibrinogen receptor CD41 and P-selectin as well as secretion of platelet-derived growth factor after platelet activation (34). Likewise, the platelet Gi protein Gα_i2_ is an essential mediator of thrombo-inflammatory organ damage in mice. This was shown in mice lacking Gα_i2_ in megakaryocytes and platelets (Gnai2^fl/fl^/PF4-Cre) that developed a dramatically reduced reperfusion injury that correlated with diminished formation of ADP-dependent platelet neutrophil complexes after myocardial IR (35).

### Na^+^/H^+^ Exchanger Isoform 1 (NHE1)

NHE1 is an integral membrane protein that removes one intracellular H^+^ for one extracellular Na^+^ protecting cells from intracellular acidification. NHE1 activation in cardiomyocytes is known to contribute to injury and arrhythmias during IR by promoting calcium overload via the sodium-calcium exchanger (60–62). But not only cardiomyocyte NHE1 activation is a driver of IRI. NHE1 is also expressed on platelets and is involved in the regulation of the platelet's intracellular pH, platelet volume as well as cell signaling and platelet activation (63, 64). In a rat model of myocardial IR the NHE1 blocker KR-32568 dose-dependently inhibited NHE-1-mediated rabbit platelet swelling and dose dependently reduced infarct size when applied 10 min before ischemia (39).

## Mediators Released by Platelets

As part of their pleiotropic actions platelets can rapidly secrete a wide array of inflammatory mediators, either from their granules or in a granule independent manner (12) which are reasonable candidates for mediating inflammation during IRI.

### Reactive Oxygen Species (ROS)

ROS occur as byproducts of certain enzymatic reactions (e.g., catalyzed by xanthine oxidases, cytochrome P450 or NADPH oxidase). ROS play important roles in cell signaling and homeostasis but are also involved in the pathogenesis of several diseases including cardiovascular disease (65, 66). In guinea pig hearts exposed to low-flow ischemia with following reperfusion activated human platelets administered in the beginning of reperfusion significantly reduced the recovery of external heart work (REHW). Coapplication of the radical scavenger enzyme superoxide dismutase improved REHW during reperfusion indicating a role of ROS in the provoked IRI. Interestingly, by coapplication of the GPIIb/IIIa-blocker tirofiban the authors could show that the platelet-induced ROS-dependent myocardial dysfunction in their experimental model was independent of intracoronary platelet adhesion (49, 56, 66). In a follow up study, by applying a platelet pretreatment with diphenyliodonium chloride Seligmann et al. elegantly proved that the shown cardiodepressive effects were mediated by ROS released from platelets and not the heart itself (49). ROS-induced effects on reperfused myocardium are based on several mechanisms including calcium overload by interference with myocardial calcium transport, damage to membranes and proteins, as well as opening of the mitochondrial permeability transition pore and subsequent apoptosis (4, 49).

### Serotonin

Serotonin (5-hydroxytryptamine) is a biogenic amine present in circulation and non-neuronal cells as peripheral hormone and in the central nervous system as neurotransmitter. In the periphery it is stored in high concentrations in dense granules of platelets. Myocardial IRI is accompanied by elevated serotonin plasma levels in mice (33). In 1994 Hohlfeld et al. demonstrated that nexopamil, a combined Ca^2+^ and serotonin antagonist, reduced infarct size and improved functional cardiac parameters in minipigs subjected to 1 h of LAD occlusion with a subsequent 3 h reperfusion. Besides calcium channel blocking activity, inhibition of ischemia-induced neutrophil activation and enhanced endogenous PGI_2_ formation were claimed to be factors contributing to the beneficial effects of nexopamil (42). Later, serotonin's harmful mode of action in IRI was partially attributed to oxidative stress caused by mitochondrial MAO-A activity. MAO-A is responsible for serotonin degradation with H_2_O_2_ production. Evidence was presented that the oxidative stress induced by this enzymatic reaction was responsible for receptor-independent apoptotic effects of serotonin in cardiomyocytes and postischemic myocardial injury (40). We found recently that absence of platelet serotonin improves outcome of mice after myocardial ischemia and reperfusion, i.e., a 30% smaller infarct size and less compromised LV function and ejection fraction, which were accompanied by reduced neutrophil infiltration within the infarcted tissue. Mechanistically, platelet-derived serotonin induced neutrophil degranulation with release of myeloperoxidase and H_2_O_2_ as well as increased surface expression of the adhesion molecule CD11b, leading to enhanced inflammation in the infarct area and reduced myocardial salvage (33).

### Platelet Activating Factor (PAF)

PAF is a phosphoglyceride produced by platelets, leukocytes and endothelial cells which acts as an autocrine and paracrine mediator on different cell types, e.g., cardiomyocytes, endothelial cells and platelets (67). During IRI high quantities of PAF (1–10 nmol/L) are released and can exert negative effects on coronary and cardiac functions, including arrhythmogenic effects (68–70). Negative effects of PAF can be mediated either by the generation of secondary mediators, or through the activation of inflammatory cells like platelets and neutrophils (67). For example, it was demonstrated that administration of a specific PAF receptor antagonist immediately before reflow in an intact sheep model reduces myocardial reperfusion injury—an impact which was partially attributed to its anti-platelet effect (44). Furthermore, PAF has been shown to stimulate NHE1 in neutrophils and platelets (63, 71). Negative consequences of NHE1 activation in the context of IRI involve platelet swelling and calcium overload in cardiomyocytes (see above). However, PAF seems to play a dual role in IR depending on its local concentration. Potential cardioprotective effects of PAF are described later in this review.

## Leukocyte-Platelet-Interactions in IRI

It has long been known that the interaction between platelets and neutrophils is associated to MI (57). Already in 1997 Neumann et al. showed that in peripheral venous blood samples of patients with AMI leukocyte platelet adhesion was increased and claimed that this was part of the regulation of the inflammatory response in acute MI (72). A causative connection between platelet neutrophil interactions and IRI was supported by several studies showing worsened cardiac functions in isolated heart models of global ischemia and reperfusion after simultaneous perfusion with both neutrophils and platelets compared to perfusion with either platelets or neutrophils alone (45, 73) as well as attenuation of these adverse effects by inhibition of platelet neutrophil complex formation (74). These relationships seem logical, especially as neutrophils interact strongly with platelets to regulate the performance of their immune cell functions (75). However, there are opposing studies as well. Seligmann et al. did not observe an additional effect of simultaneous applications of platelets and neutrophils over sole application of either of both in a study using isolated guinea pig hearts (76). In contrast, indications for a role of platelet neutrophil interactions in IRI were also found by different inhibition approaches of the P-selectin-P-selectin glycoprotein ligand 1(PSGL1) axis that led to alleviation of myocardial IRI. Several animal studies in different species demonstrated beneficial effects of P-Selectin neutralization, e.g., via small molecule inhibition (38), chemical blockade (43), or antibody blocking (77) on IRI which were all associated with an antiplatelet effect accompanied by less neutrophil infiltration or platelet-neutrophil adhesion in the infarcted region. In addition to P-selectin, also platelet GPIIb/IIIa is claimed to contribute to postischemic leukocyte adhesion (36) and also the disintegrin-dependent attenuation of platelet induced myocardial IRI was shown to be accompanied by reduced neutrophil infiltration (41).

## Platelet-Endothelial-Interactions in IRI

IR induces cellular responses on microvascular endothelial cells and circulating platelets become activated (5). At the same time the adhesion and activation of platelets goes along with the release of various proinflammatory and promitogenic substances which change chemotactic, adhesive and proteolytic properties of the endothelial cells in the local surrounding (2). The inhibition of GPVI-mediated platelet-endothelial interaction via recombinant soluble GPVI-Fc was shown to reduce platelet degranulation and the release of proinflammatory cytokines. This lead to decreased infarct size and improved cardiac function due to a reduced inflammatory response of the infarcted myocardium in a mouse model of IRI (31). Furthermore, CD40 ligand on activated platelets triggers an inflammatory reaction of endothelial cells (78) and dual neutrophil and platelet infiltration leads to enhanced P-selectin expression on the coronary microvascular endothelium in rat hearts subjected to IR (73).

## Cardioprotective Effects of Platelets in IRI

Besides the negative effects induced by platelet adhesion and aggregation as well as the platelet dependent leukocyte infiltration into the infarcted myocardium, constituents released by platelets may have beneficial effects on the integrity of the coronary endothelium and on cardiac function after IRI (79, 80). In isolated rat hearts perfusion with platelets or supernatant of aggregated platelets was shown to exhibit cardioprotective effects (46) that were partially attributed to serotonin, thromboxane A2 or adenin nucleotides and their ability to induce the release of cardiac microvascular endothelial NO and its associated tissue protecting effects (81, 82). The platelet α granule contents transforming growth factor-beta 1 (TGF-β 1) and stromal cell-derived factor (SDF)1-α were also shown to be cardioprotective (83–85)—an effect most probably mediated by enhanced cardiomyocyte proteinkinase C (PKC) activity as a prosurvival signaling mechanism (85). Also platelet-derived sphingosine-1-phosphate (S1P) seems to facilitate protection from IRI. Although platelet derived S1P can have both pro- and anti-aggregatory effects via G-protein coupled receptors on platelets it was shown to directly induce myocardial protection. S1P signals through S1P receptors of cardiomyocytes with concomitant activation of pro-survival signaling, namely the reperfusion injury salvage kinase (RISK) and the survivor activating factor enhancement (SAFE) pathway (68). SAFE and RISK were both shown to be protective on cardiomyocytes when acutely activated at the time of reperfusion, most probably through inhibition of the opening of the mitochondrial permeability transition pore (86, 87). PAF which was already mentioned as an IRI causing factor earlier in this review is cardioprotective in very low concentrations. This effect involves activation of the RISK pathway, including protein kinase C, AKT, and nitric oxide synthase (47, 67). Another described protective mechanism of platelets during IRI is the process of mitophagy which removes damaged mitochondria. Hypoxic mitophagy in platelets leads to extensive degradation of mitochondria and reduces IRI by diminishing platelet activity (5).

## Conclusions

**Figure 1:**
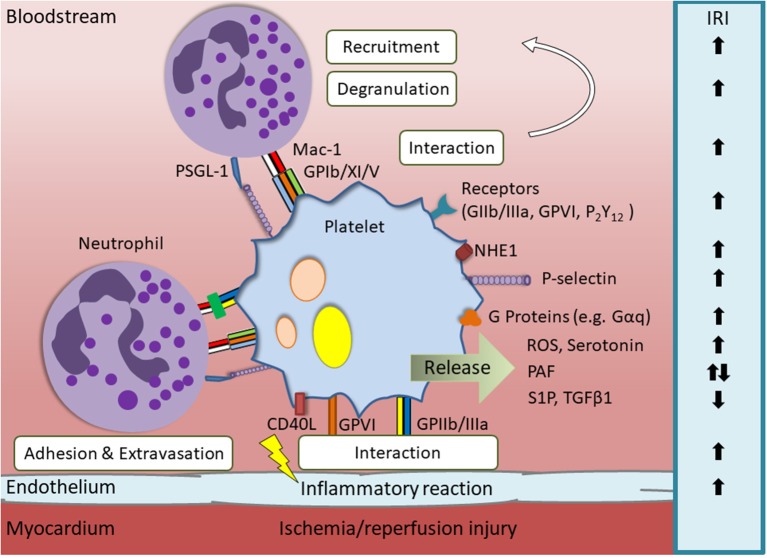
Overview of relevant platelet derived mediators and their influence on myocardial ischemia/reperfusion injury (IRI). Endothelial damage caused by IRI leads to activation of platelets. This is accompanied by upregulation of surface proteins and the release of immunomodulatory contents that influence the progression of IRI via different mechanisms. Platelet receptors that are involved in IRI aggravation are glycoprotein (GP) IIb/IIIa, GPVI and P2Y12. Additionally, platelet membrane proteins, such as sodium-hyodrogen-exchanger 1 (NHE1), Gαq, Gαi2, and P-selectin, or secretable factors, e.g., reactive oxygen species (ROS) and serotonin, worsen the cardiac outcome after myocardial infarction. Cardioprotective effects are for example exerted by platelet-derived sphingosine-1-phosphate (S1P), low concentrations of platelet activating factor (PAF) and transforming growth factor beta 1(TGFβ1).

The pathophysiology of IRI and the contribution of the involved immune cells to its progression is complex and only partially understood. However, during the past years critical roles of platelets in the origin and course of IRI as well as several underlying molecular mechanisms have been unraveled and Figure 1 summarizes most of them. Although single-agent approaches targeting these mechanisms have not yet entered clinical practice, a better understanding of platelet mechanisms in IRI could provide the basis for new and effective treatment strategies aimed at further improving protection of the myocardium during reperfusion in the future.

## Author Contributions

NS did literature research, designed figures, and wrote the first draft of the manuscript. DD contributed the idea of the manuscript, wrote sections of the manuscript, and provided critical feedback. CB supported writing and provided critical feedback. All authors contributed to manuscript revision, read, and approved the submitted version.

### Conflict of Interest Statement

The authors declare that the research was conducted in the absence of any commercial or financial relationships that could be construed as a potential conflict of interest.
